# Effect of *BRCA* mutational status on survival outcome in advanced-stage high-grade serous ovarian cancer

**DOI:** 10.1186/s13048-019-0511-7

**Published:** 2019-05-07

**Authors:** Se Ik Kim, Maria Lee, Hee Seung Kim, Hyun Hoon Chung, Jae-Weon Kim, Noh Hyun Park, Yong-Sang Song

**Affiliations:** Department of Obstetrics and Gynecology, Seoul National University College of Medicine, 101 Daehak-Ro, Jongno-Gu, Seoul, 03080 Republic of Korea

**Keywords:** Genital neoplasms, female, Ovarian neoplasms, High-grade serous carcinoma, *BRCA1/2* germline mutation, Clinical outcome, Survival outcome

## Abstract

**Objective:**

To evaluate impact of germline *BRCA* mutational status on prognosis in patients with advanced ovarian cancer.

**Methods:**

A total of 128 patients diagnosed with FIGO stage III-IV high-grade serous ovarian cancer (HGSOC) between 2008 and 2017 and underwent *BRCA1/2* gene testing at the time of or within two years from cancer diagnosis were included in this study. We compared patients’ clinicopathological characteristics and survival outcomes after primary treatment according to germline *BRCA* mutational status. Treatment-related factors that might affect patients’ survival outcome were also investigated.

**Results:**

Germline *BRCA1/2* mutations were observed in 51 women (39.8%). There were no differences in age and serum CA-125 levels at the time of HGSOC diagnosis, use of neoadjuvant chemotherapy (NAC), extent of debulking surgery, and overall survival (OS) between the *BRCA* mutation and wild-type *BRCA* groups. In contrast, the *BRCA* mutation group displayed longer progression-free survival (PFS) (median, 22.9 vs. 16.9 months, *P* = 0.001). Multivariate analyses identified germline *BRCA1/2* mutation as an independent favorable prognostic factor for PFS (adjusted HR, 0.502; 95% CI, 0.318–0.795; *P* = 0.003). In the wild-type *BRCA* group, patients who received NAC as the primary treatment had shorter PFS compared to those who received primary debulking surgery (PDS) (median, 14.2 vs. 16.9 months, *P* = 0.003). However, in the *BRCA* mutation group, PFS did not differ between the NAC and PDS groups (*P* = 0.082).

**Conclusions:**

In advanced-stage HGSOC, patients with germline *BRCA1/2* mutations have better prognosis with longer PFS than those lacking *BRCA* mutations. Prognosis after NAC was different according to the *BRCA* mutational status.

**Electronic supplementary material:**

The online version of this article (10.1186/s13048-019-0511-7) contains supplementary material, which is available to authorized users.

## Introduction

Ovarian cancer is the deadliest gynecologic malignancy, accounting for 226,000 new cases and 158,000 cancer deaths globally each year [1]. In Korea, ovarian cancer has been gradually increasing [2]. Germline mutations in *BRCA1* or *BRCA2* gene confer a high risk of developing ovarian cancer [3, 4]. A recent prospective cohort study estimated the cumulative ovarian cancer risk to age 80 years as 44% (95% confidence interval [CI], 36–53%) for *BRCA1* and 17% (95% CI, 11–25%) for *BRCA2* mutation carriers [4].

The majority (90%) of ovarian cancers are epithelial ovarian cancers (EOCs) [5]. High-grade serous ovarian cancer (HGSOC), the most prevalent and lethal form among EOCs, is of particular interest because approximately 20% of patients with this histology have germline *BRCA1/2* mutations [6]. Three recent randomized trials on maintenance therapy with poly(adenosine diphosphate)-ribose polymerase (PARP) inhibitors showed significantly improved progression-free survival (PFS) in *BRCA* mutated, platinum-sensitive relapsed HGSOC: olaparib in the SOLO-2 trial [7], rucaparib in the ARIEL3 trial [8], and niraparib in the NOVA trial [9]. The ability to identify patients with germline *BRCA1/2* mutations and evaluate their clinical outcomes are important issues in HGSOC.

To date, the exact effect of germline *BRCA1/2* mutations on ovarian cancer prognosis has not yet been determined. Several studies reported that patients with germline *BRCA* mutations have better prognosis, probably due to the high response rate to platinum-based chemotherapy [10–15]; although, other studies reported heterogeneous results [16–18]. Favorable prognosis was associated only with *BRCA2* mutated EOC, but not with *BRCA1* mutated patients [17, 18]. The study population heterogeneity and ethnicity hinder the evaluation of exact relationship between the survival outcomes and germline *BRCA1/2* mutations. Furthermore, *BRCA1* is a relatively large gene and its protein product has three representative domains, frequently mutated in cancer patients with relatively high frequency [19]. Mutations in the different domains might result in differences in cancer prognosis.

More precise knowledge regarding the effects of *BRCA* gene mutations on HGSOC prognosis and treatment method success would allow for the development of individualized treatment plans for patients with HGSOC. In addition, it is necessary to present scientific evidences on these issues in patients of Korean ethnicity. Thus, this study aimed to evaluate the effect of *BRCA* mutational status on clinical outcome in patients with advanced-stage HGSOC. We also investigated treatment-related factors that might affect HGSOC patients’ survival outcome, including use of neoadjuvant chemotherapy (NAC) and extent of debulking surgery.

## Materials and methods

This retrospective case-control study was conducted after obtaining approval from the Institutional Review Board of Seoul National University Hospital (IRB No. 1712–083-907).

### Study population

At our institutional hospital, we recommend germline *BRCA1/2* gene testing to all women with pathologically proven EOC in accordance with the position statements of the Korean Society of Gynecologic Oncology [20]. Previously, germline *BRCA1/2* gene testing was performed using direct sequencing (Sanger sequencing), including whole exon and exon-intron boundaries of *BRCA1* and *BRCA2*. However, since February 2016, next-generation sequencing (NGS) of *BRCA1* and *BRCA2* genes have been available and actively used. Germline mutations, discovered by NGS, were validated by Sanger sequencing. To determine whether the detected mutations were previously identified, we searched the Breast Cancer Information Core (BIC) database (https://research.nhgri.nih.gov/projects/bic/), the National Institutes of Health open-access database of clinically observed variants and their classification (ClinVar) (https://www.ncbi.nlm.nih.gov/clinvar/), and the previously published Korean germline *BRCA1/2* mutations [21–25]. In this study, patients with *BRCA1* or *BRCA2* gene sequence variants which were previously reported as deleterious mutations or classified as likely pathogenic or pathogenic according to the American College of Medical Genetics and Genomics and the Association for Molecular Pathology guidelines [26] were assigned to the *BRCA* mutation group. Otherwise, they were assigned to the wild-type *BRCA* group.

**Fig. 1:**
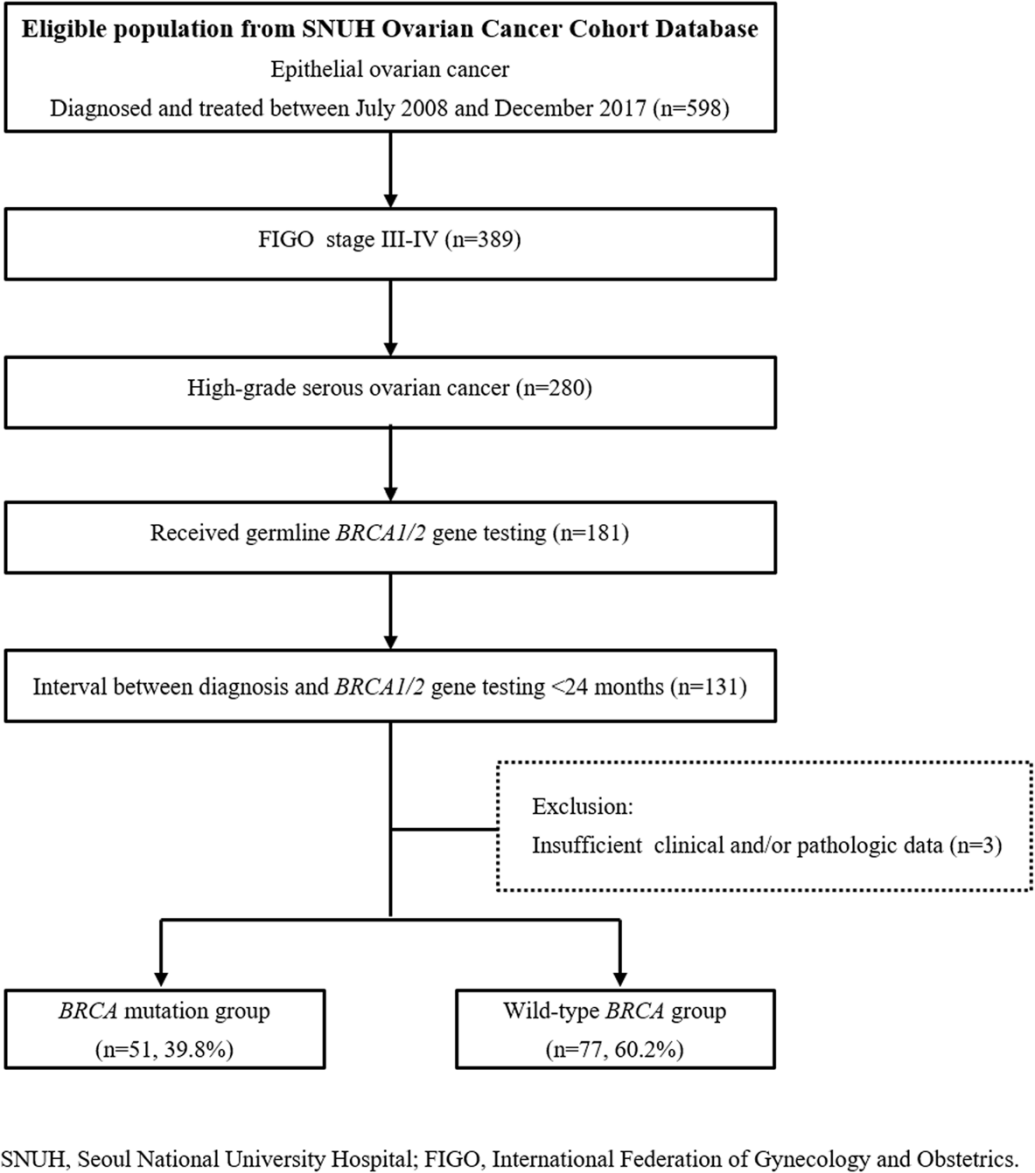
Flow diagrams depicting the selection of the study population

From our institution’s Ovarian Cancer Cohort Database, we searched relevant patients to identify those who met the following inclusion criteria: (1) diagnosed with EOC and treated between July 2008 and December 2017; (2) International Federation of Gynecology and Obstetrics (FIGO) stage III-IV disease; (3) High-grade serous histologic type; and (4) received germline *BRCA1/2* gene testing at the time of or within 2 years from cancer diagnosis. Patients with insufficient clinical and/or pathologic data were excluded from the study. Figure 1 depicts the selection of the study population. Of the 128 patients with advanced-stage HGSOC who met these criteria, 51 (39.8%) and 77 (60.2%) were included in *BRCA* mutation group and wild-type *BRCA* group, respectively. Thereafter, we compared clinicopathological characteristics and clinical outcomes between the two groups.

Three representative domains of the BRCA1 protein are as follows: 1) the N-terminal Really Interesting New Gene (RING) domain (exons 2–7); 2) Exons 11–13, that covers over 65% of the sequence of *BRCA1*; and 3) the BRCA1 C-terminal (BRCT) domain (exons 16–24) [19]. Considering these three domains, 37 patients of the *BRCA* mutation group were divided into three subgroups depending on the position of *BRCA1* mutations, and their survival outcomes were compared.

### Data collection

We reviewed medical records to collect information about clinicopathological characteristics, such as age and serum CA-125 levels at diagnosis, and FIGO stage, and primary treatment of EOC, such as use of NAC and extent of residual tumour after debulking surgery. Optimal debulking was defined when the maximal diameter of residual tumour was less than 1 cm. Whether the patients received NAC followed by interval debulking surgery or primary debulking surgery (PDS), all patients received post-operative adjuvant taxane- and platinum-based chemotherapy as the primary treatment. However, none of them received any PARP inhibitors. Patients’ personal and familial histories at the time of HGSOC diagnosis were retrieved from medical records. We collected the patients’ parity, menstruation status, personal and familial history of breast cancer and ovarian cancer, and the number of relatives (up to second-degree family members) who had a history of breast or ovarian cancers.

PFS was calculated as the time interval between the date of initial diagnosis and the date of disease progression using the Response Evaluation Criteria in Solid Tumours version 1.1 [27]. Treatment-free interval (TFI) was calculated as the time interval between the date of completion of primary treatment and the date of disease progression. Platinum-sensitive recurrence (PSR) was defined when the TFI was 6 months or longer, whereas platinum-resistant recurrence (PRR) was defined when the TFI was shorter than 6 months. Overall survival (OS) was calculated as the time interval between the date of initial diagnosis to the date of cancer-related death or end of the study.

### Statistical analysis

Differences in clinicopathological characteristics were evaluated between the two groups: Student’s *t*-test and Mann-Whitney *U*-test were used to compare continuous variables, and Pearson’s chi-squared test and Fisher’s exact test were used to compare categorical variables. The Kaplan-Meier methods with log-rank test were used for survival analyses. Hazard ratios (HRs) and 95% CIs were calculated using Cox proportional hazards regression models. We used SPSS software (version 21.0; SPSS Inc., Chicago, IL, USA) for these analyses. All statistical tests were two-sided, and a *P* value < 0.05 was considered statistically significant.

## Results

### Characteristics of study population

The patients’ clinicopathological characteristics are presented in Table 1. No differences in age and serum CA-125 levels at the time of HGSOC diagnosis, use of NAC, and rates of optimal debulking surgery were observed between the *BRCA* mutation group and the wild-type *BRCA* group. The time interval between diagnosis and genetic test was not different either. Distributions of FIGO stage IIIA1 to IVB were not different between the two groups (*P* = 0.077). However, FIGO stage IV disease were less common in the patients who have *BRCA* mutation compared to those with *BRCA* wild-type (25.5% vs. 49.4%, *P* = 0.007). In the *BRCA* mutation group, 37 (72.5%) and 14 (27.5%) patients had mutation in *BRCA1* or *BRCA2*, respectively; no patients had double mutations.

**Table 1:** Clinicopathological characteristics of patients at diagnosis of epithelial ovarian cancer

Characteristics	All (*n*=128, %)	*BRCA* mutation (*n*=51, %)	*BRCA* wild-type (*n*=77, %)	*P*
Age, years				
Mean ± SD	56.4 ± 10.3	54.7 ± 9.9	57.5 ± 10.4	0.130
<50	39 (30.5)	20 (39.2)	19 (24.7)	0.080
≥50	89 (69.5)	31 (60.8)	58 (75.3)	
BMI, kg/m2				
Mean ± SD	23.4 ± 3.5	23.2 ± 3.7	23.6 ± 3.5	0.576
CA-125, IU/ml				
Median (range)	921.5 (13.0−10000.0)	795.0 (34.9−9926.0)	1296.0 (13.0−10000.0)	0.724
FIGO stage				0.077
III	77 (60.2)	38 (74.5)	39 (50.6)	0.007
IIIA1	4 (3.1)	3 (5.9)	1 (1.3)	
IIIA2	4 (3.1)	1 (2.0)	3 (3.9)	
IIIB	12 (9.4)	5 (9.8)	7 (9.1)	
IIIC	57 (44.5)	29 (56.9)	28 (36.4)	
IV	51 (39.8)	13 (25.5)	38 (49.4)	0.007
IVA	6 (4.7)	1 (2.0)	5 (6.5)	
IVB	45 (35.2)	12 (23.5)	33 (42.9)	
Primary treatment strategy				0.846
PDS	79 (61.7)	32 (62.7)	47 (61.0)	
NAC	49 (38.3)	19 (37.3)	30 (39.0)	
Residual tumor after PDS/IDS				0.192
Optimal debulking	109 (85.2)	46 (90.2)	63 (81.8)	
Suboptimal debulking	19 (14.8)	5 (9.8)	14 (18.2)	
Recurrence				
No	39 (30.5)	20 (39.2)	19 (24.7)	0.080
Yes	89 (69.5)	31 (60.8)	58 (75.3)	
Platinum-sensitive recurrence	62 (48.4)	25 (49.0)	37 (48.1)	0.099
Platinum-resistant recurrence	27 (21.1)	6 (11.8)	21 (27.3)	
*BRCA* mutation				
*BRCA1*	37 (28.9)	37 (72.5)		
*BRCA2*	14 (10.9)	14 (27.5)		
Both	0 (0)	0 (0)		
Interval between diagnosis and genetic test, months				
Median (range)	3.5 (0−22.7)	2.6 (0−22.4)	4.3 (0.1−22.7)	0.065

*Abbreviations: BMI* body mass index, *CA-125* cancer antigen 125, *FIGO* International Federation of Gynecology and Obstetrics, *PDS* primary debulking surgery, *NAC* neoadjuvant chemotherapy, *IDS* interval debulking surgery, *SD* standard deviation

The patients’ personal and familial histories are presented in Additional file 1: Table S1. There were no differences in parity, menstruation status, and familial history of ovarian cancer between the two groups. However, patients with *BRCA* mutations had significantly higher personal history of breast cancer (23.5% vs. 7.8%, *P* = 0.012) and familial history of breast cancer (27.5% vs. 2.6%, *P* < 0.001) than those in the wild-type *BRCA* group. The number of relatives with breast cancer was also higher in the *BRCA* mutation group (*P <* 0.001).

Additional file 1: Table S2 depicts clinicopathological characteristics of patients according to the primary treatment strategy. In the *BRCA* mutation group, age and serum CA-125 levels at diagnosis, and residual tumour after surgery were similar between patients who received PDS and those with NAC. However, FIGO stage IV disease were more frequent in patients with NAC (47.4% vs. 12.5%, *P* = 0.009). In the wild-type *BRCA* group, patients with NAC had higher initial serum CA-125 levels (median, 1946.0 vs. 764.0, *P* = 0.011) and showed a trend towards more FIGO stage IV disease (63.3% vs. 40.4%, *P* = 0.050), compared to those with PDS. However, proportions of patients who achieved optimal debulking surgery were not different; 86.7 and 78.7% of patients who received NAC and PDS, respectively (*P* = 0.378).

### Comparisons of survival outcome between the *BRCA *mutation and wild-type *BRCA* groups

The median observation period was 26.3 months (range, 8.1–94.4 months). During this time, 31 patients (60.8%) in the *BRCA* mutation group and 58 patients (75.3%) in the wild-type *BRCA* group experienced disease recurrence. Among them, the median TFI was longer in the patients with *BRCA* mutations (12.3 months vs. 9.0 months, *P* = 0.002). However, the proportions of those with PSRs among the recurred were similar between the two groups (80.6% vs. 63.8%, *P* = 0.099).

**Fig. 2:**
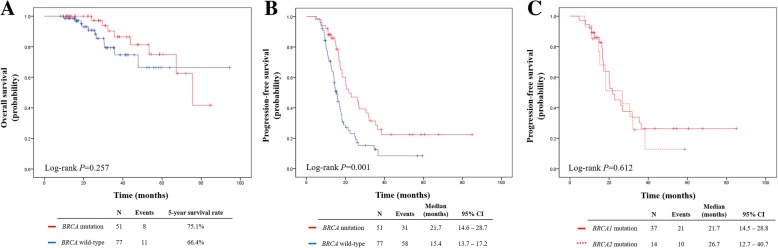
Survival outcomes of *BRCA* mutation and wild-type *BRCA* groups. **a** Overall survival. **b** Progression-free survival. **c** Progression-free survival according to the mutated *BRCA* gene

Survival outcomes for the *BRCA* mutation and wild-type *BRCA* groups are presented in Fig. 2. There were no significant differences in OS between the two groups (5-year survival rates, 75.1% vs. 66.4%, *P* = 0.257). By contrast, patients in the *BRCA* mutation group had significantly longer PFS than those in the wild-type *BRCA* group (median, 21.7 vs. 15.4 months, *P* = 0.001). In terms of specific *BRCA* gene type, the patients who had *BRCA1* mutation and those who had *BRCA2* mutation showed no differences in PFS (median, 21.7 vs. 26.7 months, *P* = 0.612).

Multivariate analyses adjusting age, FIGO stage, primary treatment strategy, residual tumour after debulking surgery, and *BRCA* mutational status revealed only NAC (compared to PDS) as an independent poor prognostic factor for OS (adjusted HR, 4.098; 95% CI, 1.478–11.359; *P* = 0.007) (Table 2). Meanwhile, the *BRCA1/2* mutation was identified as an independent favorable prognostic factor for PFS (adjusted HR, 0.502; 95% CI, 0.318–0.795; *P* = 0.003), and NAC was also associated with poor PFS (adjusted HR, 2.103; 95% CI, 1.321–3.348; *P* = 0.002) (Table 3).

**Table 2:** Factors associated with overall survival

Characteristics	*N*	Univariate analysis			Multivariate analysis		
		HR	95% CI	*P*	Adjusted HR	95% CI	*P*
Age, years							
<50	39	1 (Ref)	−	−	1 (Ref)	−	−
≥50	89	2.848	0.825−9.835	0.098	2.407	0.668−8.670	0.179
FIGO stage							
III	77	1 (Ref)	−	−	1 (Ref)	−	−
IV	51	2.055	0.734−5.753	0.170	1.248	0.434−3.591	0.682
Primary treatment strategy							
PDS	79	1 (Ref)	−	−	1 (Ref)	−	−
NAC	49	3.790	1.483−9.691	0.005	4.098	1.478−11.359	0.007
Residual tumor after PDS/IDS							
Optimal debulking	109	1 (Ref)	−	−	1 (Ref)	−	−
Suboptimal debulking	19	1.723	0.545−5.442	0.354	1.935	0.559−6.703	0.298
*BRCA* status							
Wild-type	77	1 (Ref)	−	−	1 (Ref)	−	−
Mutation	51	0.584	0.228−1.495	0.262	0.768	0.283−2.082	0.603

*Abbreviations: CA-125* cancer antigen 125, *FIGO* International Federation of Gynecology and Obstetrics, *PDS* primary debulking surgery, *NAC* neoadjuvant chemotherapy, *IDS* interval debulking surgery, *HR* hazard ratio, *CI* confidence interval, *Ref* reference

**Table 3:** Factors associated with progression-free survival

Characteristics	*N*	Univariate analysis			Multivariate analysis		
		HR	95% CI	*P*	Adjusted HR	95% CI	*P*
Age, years							
<50	39	1 (Ref)	−	−	1 (Ref)	−	−
≥50	89	1.540	0.958−2.477	0.075	1.377	0.850−2.232	0.194
FIGO stage							
III	77	1 (Ref)	−	−	1 (Ref)	−	−
IV	51	1.903	1.244−2.912	0.003	1.358	0.867−2.126	0.182
Primary treatment strategy							
PDS	79	1 (Ref)	−	−	1 (Ref)	−	−
NAC	49	2.098	1.373−3.206	0.001	2.103	1.321−3.348	0.002
Residual tumor after PDS/IDS							
Optimal debulking	109	1 (Ref)	−	−	1 (Ref)	−	−
Suboptimal debulking	19	1.515	0.867−2.648	0.145	1.587	0.879−2.865	0.126
*BRCA* status							
Wild-type	77	1 (Ref)	−	−	1 (Ref)	−	−
Mutation	51	0.484	0.310−0.755	0.001	0.502	0.318−0.795	0.003

*Abbreviations: CA-125* cancer antigen 125, *FIGO* International Federation of Gynecology and Obstetrics, *PDS* primary debulking surgery, *NAC* neoadjuvant chemotherapy, *IDS* interval debulking surgery, *HR* hazard ratio, *CI* confidence interval, *Ref* reference

### Comparisons of survival outcome according to the primary treatment strategy

**Fig. 3:**
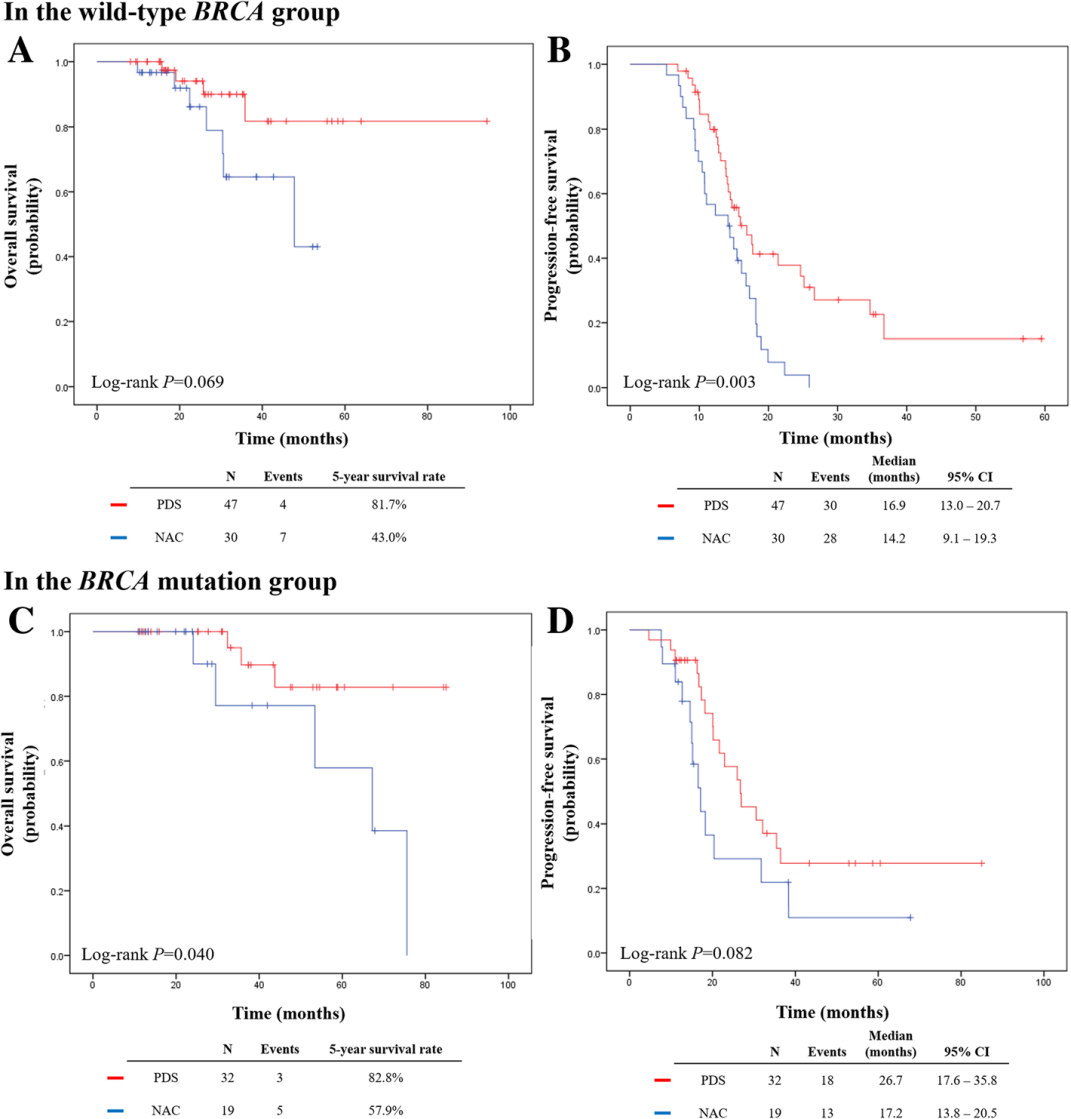
Comparisons of survival outcomes in the wild-type *BRCA* group (upper) and in the *BRCA* mutation group (lower) according to the primary treatment strategy. **a**, **c** Overall survival. **b**, **d** Progression-free survival

Next, we compared the survival outcomes in all patients according to the primary treatment strategy (Additional file 1: Figure S1). Patients who received NAC had significantly poorer survival outcomes than those who received PDS (OS, *P* = 0.003; and PFS, *P* < 0.001). We performed subgroup analyses considering *BRCA* mutational status. In the wild-type *BRCA* group, compared to PDS, patients who received NAC had poorer PFS (median, 14.2 vs. 16.9 months, *P* = 0.003), whereas no differences in OS were observed (Fig. 3 a, b). In the *BRCA* mutation group, patients who received NAC showed poorer OS (5-year survival rates, 57.9% vs. 82.8%; *P* = 0.040). However, PFS was not different between NAC and PDS treatments (median, 17.2 vs. 26.7 months; *P* = 0.082) (Fig. 3 c, d).

Lastly, we also performed subgroup analyses limited to the specific primary treatment strategy. In PDS group, multivariate analyses revealed that *BRCA* mutational status did not affect both OS and PFS (Additional file 1: Table S3). However, in NAC group, the *BRCA1/2* mutation was identified as an independent favorable prognostic factor for PFS (adjusted HR, 0.433; 95% CI, 0.202–0.926; *P* = 0.031). Suboptimal deublking was a poor prognostic factor for PFS (adjusted HR, 3.753; 95% CI, 1.294–10.890; *P* = 0.015) (Additional file 1: Table S4).

### Comparisons of survival outcome according to the position of *BRCA1* gene mutation

Detailed deleterious *BRCA1* gene mutations of 37 patients in the *BRCA* mutation group are displayed in Additional file 1: Table S5. The most frequently mutated domain was exons 11–13, in which 24 (64.9%) patients were included. Of the others, 6 (16.2%) and 7 (18.9%) patients had mutations in the N-terminal RING domain and BRCT domain, respectively. All 6 mutations in the N-terminal RING domain were the same nonsense mutation. In survival analysis, the three subgroups showed similar OS (*P* = 0.643) and PFS (*P* = 0.963) (Additional file 1: Figure S2).

## Discussion

This study analysed correlations between *BRCA* mutational status and clinical outcome in patients with advanced-stage HGSOC. Patients with germline *BRCA1/2* mutations had better prognosis with longer PFS than those with wild-type *BRCA1/2* genes. In terms of specific *BRCA* gene type, *BRCA1* mutation and *BRCA2* mutation showed no differences in PFS.

In Korea, the National Health Insurance System approved and started to cover *BRCA1/2* gene testing for patients with EOC. In addition, we recommend germline *BRCA1/2* gene testing to all women with pathologically proven EOC at our institutional hospital. Nevertheless, real-world uptake rate of the gene testing was less than 70% in our hospital: Of 280 patients diagnosed with FIGO stage III-IV HGSOC, 181 patients received germline *BRCA1/2* gene testing (64.6%). A high cost and cultural factors, such as social stigma and guilty feelings to familial members, might hinder patients from germline gene testing. It is obvious that the longer patients survive, the more they tend to get tested. Thus, we confined the study population to those who received germline *BRCA1/2* gene testing at the time of or within 2 years from cancer diagnosis to minimize survival bias.

Previous studies evaluated the effects of germline *BRCA1/2* mutations on EOC prognosis. Some studies reported that only OS, not PFS, was significantly longer in the *BRCA* mutation group compared to the wild-type *BRCA* group [10–12]. Other studies reported that both OS and PFS were significantly improved in the *BRCA* mutation group [6, 14, 15]. An Israeli nationwide study reported improved long-term survival in *BRCA1/2* mutation carriers [13]. Analyses of The Cancer Genome Atlas project revealed that *BRCA2* mutation, but not *BRCA1* mutation, was associated with significantly improved OS and PFS [18]. Herein, our study provides further evidence that *BRCA* mutation is associated with improved PFS. We admit the proportion of patients with stage IV was significantly higher in the *BRCA* wild-type group, compared with the *BRCA* mutation group. Suboptimal debulking surgery was more common in the *BRCA* wild-type group without statistical significance. This might influence on better survival outcome in the *BRCA* mutation group. However, we performed multivariate analyses adjusting these factors, and concluded that *BRCA* mutational status significantly affects patients’ survival outcome.

Better survival outcome of HGSOC with germline *BRCA1/2* mutation is probably due to distinct clinical features and a high response rate to platinum-based chemotherapy. Both *BRCA1* and *BRCA2* are tumour suppressor genes, and their functioning proteins have major roles in DNA double-strand break repair through homologous recombination (HR) [28–30]. In the absence of functional *BRCA1/2* genes, HGSOC have unstable genomes that are deficient in HR repair. This causes increased sensitivity to DNA-damaging chemotherapeutic agents, which is known as synthetic lethality [31]. In the current study, none of the patients received PARP inhibitors (e.g., olaparib), which are proven to increase PFS in patients with *BRCA*-mutated, platinum-sensitive relapsed ovarian cancer. Therefore, we believe that our study results show the relatively pure effect of *BRCA* mutational status on survival outcome in advanced-stage HGSOC.

In fact, the *BRCA1* gene is a large gene with 24 exons encoding a protein of 1863 amino acids. The N-terminal RING domain is an important element of ubiquitin E3 ligases, which catalyze protein ubiquitination [32], and the BRCT domain is essential for repair of DNA [33]. Each functional domains are known to have selected binding partners [34]. Up to our knowledge, little is known about the clinical effects of differently mutated *BRCA1* domains on prognosis of HGSOC. However, differences in survival outcomes were not observed according to the position of *BRCA1* gene in this study; small sample size might hinder the exact impact. Therefore, further large studies are warranted.

In the current study, patients who received NAC showed significantly poorer OS and PFS than those who received PDS (*P* = 0.003 and *P* < 0.001, respectively). This might originate from gynecologic oncologists’ preference to PDS at our institution. To ellucidate whether patients with *BRCA1/2* mutations have more favorable responses to NAC and better survival outcomes or not, we compared survival outcomes among 49 patients who received NAC according to the *BRCA* mutational status. While no difference in OS was observed between the *BRCA* mutation and wild-type *BRCA* groups (median, 67.2 and 47.8 months, *P* = 0.231), patients with germline *BRCA* mutations had improved PFS (median, 17.2 and 14.2 months, *P* = 0.014). Multivariate analyses revealed that the *BRCA1/2* mutation was an independent favorable prognostic factor for PFS. These results are similar to those of a recent multi-institutional study [35]. However, its study design was quite different: the authors did not confine the study population to the specific histologic type of EOC and performed three-group comparisons; patients with germline *BRCA1/2* mutations, patients without germline *BRCA1/2* mutations, and patients with no genetic testing.

Furthermore, we compared patients’ survival outcomes according to the primary treatment strategy in the wild-type *BRCA* group. Despite of no differences in characteristics such as FIGO stage and residual tumour after surgery, patients who received NAC had significantly poorer PFS than those who received PDS (median, 14.2 vs. 16.9 months, *P* = 0.003). Similar results were also reported in a previous retrospective multicenter study of Petrillo et al. [36]. In the *BRCA1/2* mutation group, although stage IV disease were more frequent in NAC group, PFS did not differ between the NAC and PDS groups (*P* = 0.082). However, the NAC group showed significantly poorer OS (5-year survival rates, 57.9% vs. 82.8%; *P* = 0.040).

The *BRCA* mutational status might differentially affect survival outcomes after different primary treatment strategies due to different initial disease patterns of HGSOC and different responses to chemotherapy. HGSOC patients with *BRCA1/2* mutations had significantly higher peritoneal tumour load and significantly increased frequency of bulky lymph nodes at diagnosis than those with wild-type *BRCA* genes [36]. A recent retrospective study also reported that nodular peritoneal disease pattern was significantly associated with *BRCA* mutations, whereas mesenteric involvement and supradiaphragmatic lymphadenopathy were significantly associated with wild-type *BRCA* genes [37]. Although we did not evaluate initial disease patterns, these features might have affected physician’s selection of a primary treatment strategy. Nevertheless, the high response rate to platinum-based chemotherapy in patients with *BRCA1/2* mutations might have similarly affected both NAC and PDS cases, leading to no observable differences in PFS.

Ovarian cancers are known to develop in younger women with germline *BRCA1/2* mutations than otherwise. In the current study, mean age at HGSOC diagnosis for those with *BRCA* mutations was approximately 3 years younger than for those with wild-type *BRCA* genes, however, without statistical significance (*P* = 0.130). We also observed that patients in *BRCA* mutation group had significantly higher personal history and family history of breast cancers reflecting the fact that mutations in the *BRCA1/2* genes is the most common cause of hereditary forms of both breast and ovarian cancer.

Currently, the National Comprehensive Cancer Network guidelines recommend that all women with epithelial ovarian, fallopian tube, and primary peritoneal cancers be referred for genetic risk evaluation and be subjected to *BRCA1/2* gene testing [38]. In addition to this, we suggest that *BRCA1/2* gene testing should be performed as soon as possible after EOC has been diagnosed. Because prediction of cancer prognosis and implementation of individualized treatment (e.g., assignment of the patients to PDS or NAC as primary treatment or administration of maintenance PARP inhibitors) would be facilitated based on the early genetic test results. However, in the same way as now, it is difficult in reality. For example, the median time interval between EOC diagnosis and genetic test was 3.5 months in this study, which was too late to determine patients’ primary treatment strategy. Time required for *BRCA1/2* gene sequencing and analysis itself should be also shortened considerably. We should change the way we do it now.

This study has several limitations. First, selection bias and other issues may be present due to the retrospective study design. Second, the sample size and death events might be insufficient to properly assess OS. Third, only the primary treatment was investigated in detail. Nevertheless, in our current study, the study population was more specific than in previous studies: only the patients with advanced-stage HGSOC were selected. We also tried to minimize survival bias by confining the study population to those who received germline *BRCA1/2* gene testing less than 2 years from initial diagnosis. From the clearly defined methods, our study results provide valuable information that can be applied in clinical practice.

## Conclusions

In conclusion, we identified germline *BRCA1/2* mutation as a prognostic factor to improve survival outcomes in advanced-stage HGSOC. We also provide evidence that *BRCA* mutational status has a major influence on HGSOC prognosis. *BRCA1/2* gene testing might be a useful tool to provide individualized treatment. For HGSOC patients with wild-type *BRCA*, PDS appears to be a better choice for primary treatment than NAC. In contrast, PFS did not differ according to the primary treatment strategy for patients with germline *BRCA1/2* mutations. These results require validation in larger prospective cohort studies.

## Additional file

Additional file 1: **Figure S1.** Comparisons of survival outcomes for all patients according to the primary treatment strategy. (A) Overall survival. (B) Progression-free survival. **Figure S2.** Comparisons of survival outcomes among the patients with germline *BRCA1* mutations according to the three domains: 1) the N-terminal Really Interesting New Gene (RING) domain (exons 2–7); 2) Exons 11–13, that covers over 65% of the sequence of *BRCA1*; and 3) the BRCA1 C-terminal (BRCT) domain (exons 16–24). (A) Overall survival. (B) Progression-free survival. **Table S1.** Personal and familial histories of patients at diagnosis of epithelial ovarian cancer. **Table S2.** Clinicopathological characteristics of patients according to the primary treatment strategy. **Table S3.** Factors associated with survival outcomes in primary debulking surgery group. **Table S4.** Factors associated with survival outcomes in neoadjuvant chemotherapy group. **Table S5.** Deleterious *BRCA1* gene mutations in this study. (ZIP 580 kb)

## Abbreviations

EOC: Epithelial ovarian cancer
FIGO: International Federation of Gynecology and Obstetrics
HGSOC: High-grade serous ovarian cancer
NAC: Neoadjuvant chemotherapy
NGS: Next-generation sequencing
OS: Overall survival
PARP: Poly(adenosine diphosphate)-ribose polymerase
PDS: Primary debulking surgery
PFS: Progression-free survival
PRR: Platinum-resistant recurrence
PSR: Platinum-sensitive recurrence
TFI: Treatment-free interval
