# Supplementary material for: Effect of BRCA mutational status on survival outcome in advanced-stage high-grade serous ovarian cancer
Source: J Ovarian Res. 2019 May 7;12:40. doi: 10.1186/s13048-019-0511-7 (PMC6505247; doi:10.1186/s13048-019-0511-7)
Supplement: Supplementary file 1 — Figure S1. Comparisons of survival outcomes for all patients according to the primary treatment strategy. (A) Overall survival. (B) Progression-free survival. Figure S2. Comparisons of survival outcomes among the patients with germline BRCA1 mutations according to the three domains: 1) the N-terminal Really Interesting New Gene (RING) domain (exons 2–7); 2) Exons 11–13, that covers over 65% of the sequence of BRCA1; and 3) the BRCA1 C-terminal (BRCT) domain (exons 16–24). (A) Overall survival. (B) Progression-free survival. Table S1. Personal and familial histories of patients at diagnosis of epithelial ovarian cancer. Table S2. Clinicopathological characteristics of patients according to the primary treatment strategy. Table S3. Factors associated with survival outcomes in primary debulking surgery group. Table S4. Factors associated with survival outcomes in neoadjuvant chemotherapy group. Table S5. Deleterious BRCA1 gene mutations in this study. (ZIP 580 kb) [file 13048_2019_511_MOESM1_ESM.zip › New Supplementary Files.pdf]

Supplementary Figure 1.

(A)

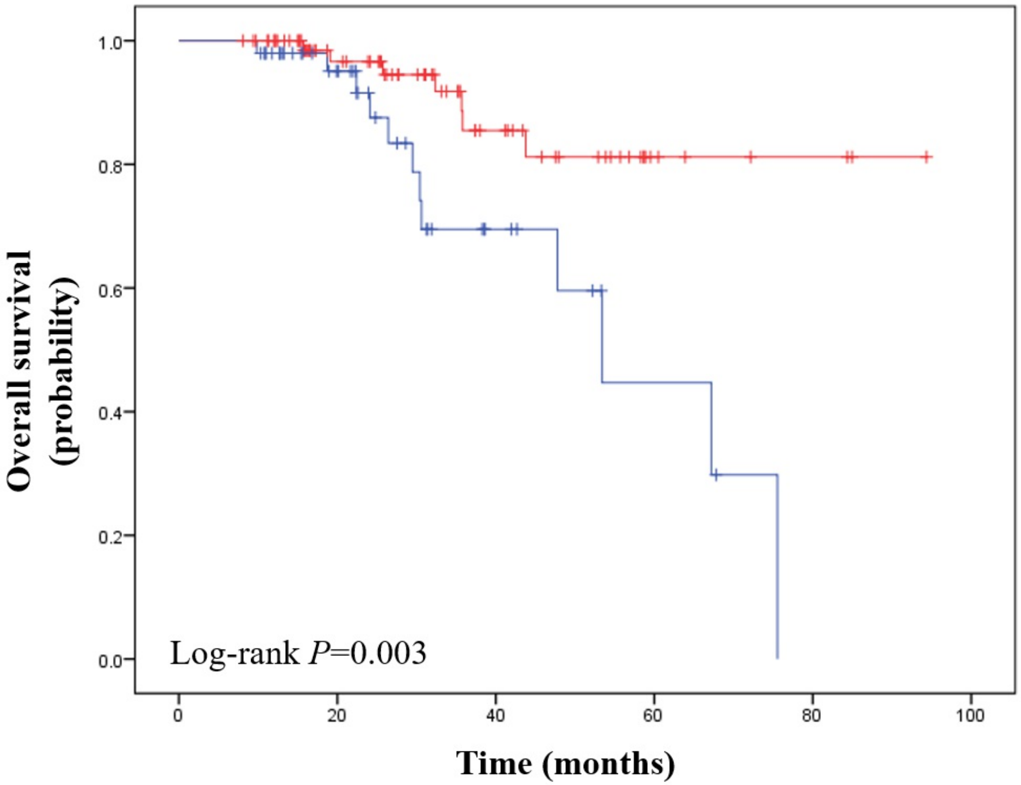

	N	Events	5-year survival rate
PDS	79	7	81.2%
NAC	49	12	44.7%

(B)

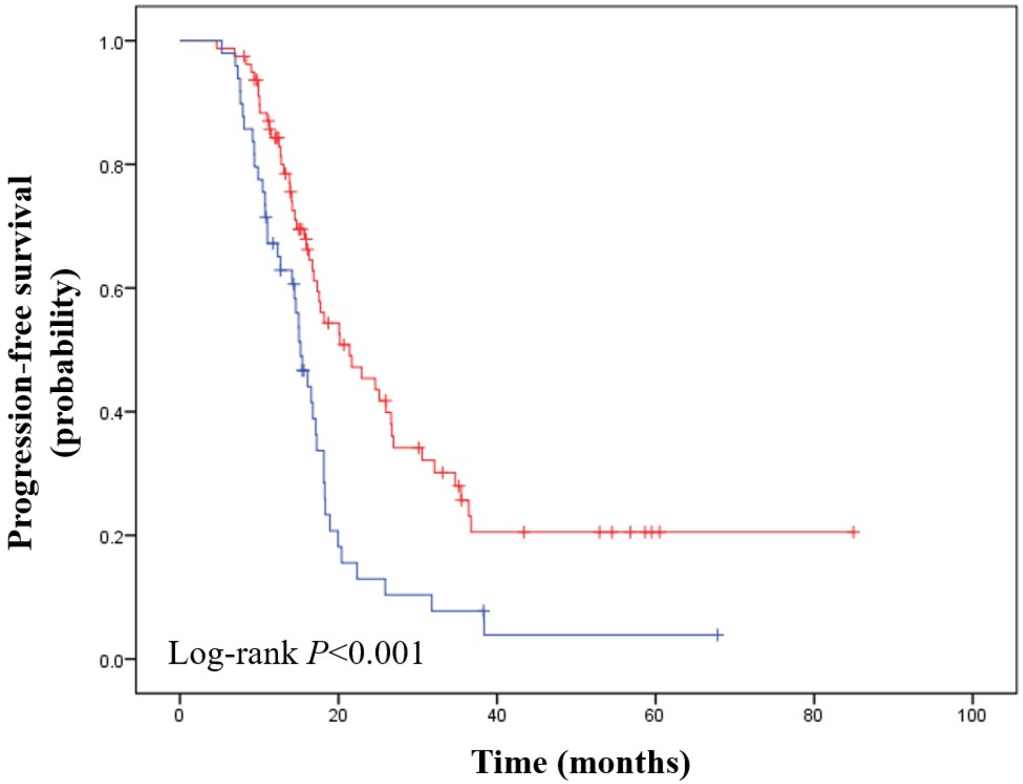

	N	Events	Median (months)	95% CI
PDS	79	48	21.4	14.5 – 28.3
NAC	49	41	15.2	13.4 – 17.0

Supplementary Figure 2.

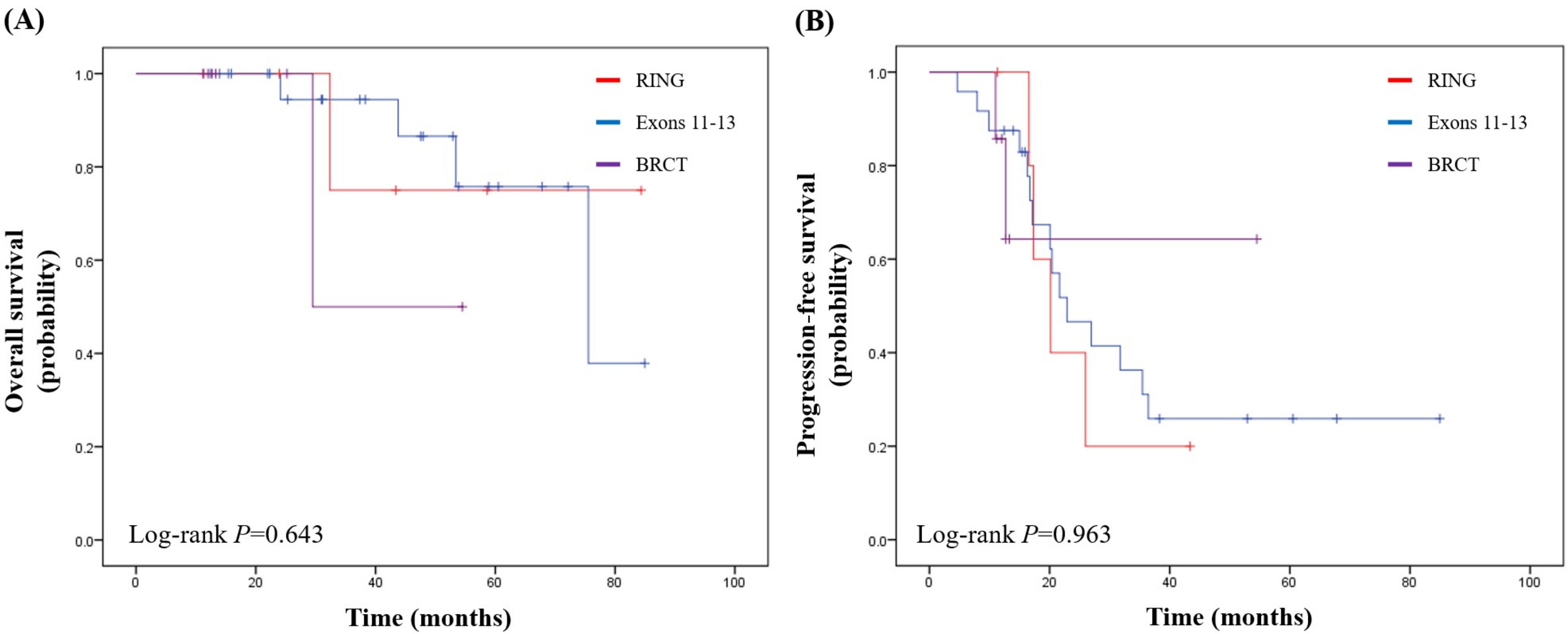

Supplementary Table 1. Personal and familial histories of patients at diagnosis of epithelial ovarian cancer

Characteristics	All (*n*=128, %)	*BRCA* mutation (*n*=51, %)	*BRCA* wild-type (*n*=77, %)	*P*
Parity				
Mean ± SD	2.2 ± 1.2	2.1 ± 1.1	2.2 ± 1.2	0.815
Nulliparity	12 (9.4)	5 (9.8)	7 (9.1)	1.000
Amenorrhea[a)]				0.792
No	36 (28.1)	15 (29.4)	21 (27.3)	
Yes	92 (71.9)	36 (70.6)	56 (72.7)	
Personal history of breast cancer	18 (14.1)	12 (23.5)	6 (7.8)	0.012
Early onset of breast cancer [b)]	3 (2.3)	2 (3.9)	1 (1.3)	0.563
Bilateral breast cancer	1 (0.8)	1 (2.0)	0	0.398
Family history of breast cancer	16 (12.5)	14 (27.5)	2 (2.6)	<0.001
No. of relatives with breast cancer				
Mean (range)	0.14 (0–2)	0.29 (0–2)	0.04 (0–2)	<0.001
Family history of ovarian cancer	4 (3.1)	3 (5.9)	1 (1.3)	0.301
No. of relatives with ovarian cancer				
Mean (range)	0.04 (0–2)	0.08 (0–2)	0.01 (0–1)	0.263

[a)] Amenorrhea was defined as the absence of menstruation for at least 6 months at diagnosis.
[b)] Early onset is diagnosed at 40 years of age or younger.

Supplementary Table 2. Clinicopathological characteristics of patients according to the primary treatment strategy

Characteristics	*BRCA* mutation			*BRCA* wild-type		
	PDS (*n*=32, %)	NAC (*n*=19, %)	*P*	PDS (*n*=47, %)	NAC (*n*=30, %)	*P*
Age, years						
Mean ± SD	54.6 ± 10.5	54.8 ± 9.2	0.966	56.1 ± 9.5	59.7 ± 11.6	0.143
BMI, kg/m2						
Mean ± SD	23.2 ± 3.8	23.1 ± 3.7	0.948	23.6 ± 3.2	23.5 ± 3.8	0.901
CA-125, IU/ml						
Median (range)	695.5 (34.9-9926.0)	1390.0 (194.0-7846.0)	0.119	764.0 (38.1-4452.0)	1946.0 (13.0-10000.0)	0.011
FIGO stage			0.009			0.050
III	28 (87.5)	10 (52.6)		28 (59.6)	11 (36.7)	
IV	4 (12.5)	9 (47.4)		19 (40.4)	19 (63.3)	
Residual tumor after PDS/IDS			0.639			0.378
Optimal debulking	28 (87.5)	18 (94.7)		37 (78.7)	26 (86.7)	
Suboptimal debulking	4 (12.5)	1 (5.3)		10 (21.3)	4 (13.3)	

Abbreviations: BMI, body mass index; CA-125, cancer antigen 125; FIGO, International Federation of Gynecology and Obstetrics; PDS, primary debulking surgery; NAC, neoadjuvant chemotherapy; IDS, interval debulking surgery; SD, standard deviation.

Supplementary Table 3. Factors associated with survival outcomes in primary debulking surgery group

Characteristics	*N*	Overall survival			Progression-free survival		
		Adjusted HR	95% CI	*P*	Adjusted HR	95% CI	*P*
Age, years							
<50	27	1 (Ref)	–	–	1 (Ref)	–	–
≥50	52	3.061	0.355–26.418	0.309	1.429	0.757–2.701	0.271
FIGO stage							
III	56	1 (Ref)	–	–	1 (Ref)	–	–
IV	23	1.296	0.232–7.254	0.768	1.605	0.872–2.953	0.129
Residual tumor after PDS							
Optimal debulking	65	1 (Ref)	–	–	1 (Ref)	–	–
Suboptimal debulking	14	1.939	0.355–10.584	0.444	1.173	0.575–2.393	0.662
BRCA status							
Wild-type	47	1 (Ref)	–	–	1 (Ref)	–	–
Mutation	32	0.619	0.131–2.912	0.543	0.554	0.300–1.024	0.059

Abbreviations: CA-125, cancer antigen 125; FIGO, International Federation of Gynecology and Obstetrics; HR, hazard ratio; CI, confidence interval; Ref, reference.

Supplementary Table 4. Factors associated with survival outcomes in neoadjuvant chemotherapy group

Characteristics	*N*	Overall survival			Progression-free survival		
		Adjusted HR	95% CI	*P*	Adjusted HR	95% CI	*P*
Age, years							
<50	12	1 (Ref)	–	–	1 (Ref)	–	–
≥50	37	4.745	0.552–40.788	0.156	1.053	0.469–2.365	0.900
FIGO stage							
III	21	1 (Ref)	–	–	1 (Ref)	–	–
IV	28	1.328	0.330–5.347	0.690	1.066	0.547–2.078	0.852
Residual tumor after IDS							
Optimal debulking	44	1 (Ref)	–	–	1 (Ref)	–	–
Suboptimal debulking	5	2.486	0.469–13.189	0.285	3.753	1.294–10.890	0.015
BRCA status							
Wild-type	30	1 (Ref)	–	–	1 (Ref)	–	–
Mutation	19	0.630	0.121–3.275	0.583	0.433	0.202–0.926	0.031

Abbreviations: CA-125, cancer antigen 125; FIGO, International Federation of Gynecology and Obstetrics; IDS, interval debulking surgery; HR, hazard ratio; CI, confidence interval; Ref, reference.

Supplementary Table 5. Deleterious *BRCA1* gene mutations in this study

Exon	BIC nomenclature	HGVS Nucleotide change	Protein change	Mutation type	*n*
7	509C>A	c.390C>A	p.Tyr130*	Nonsense	6
11	1041_1043delAGCinsT	c.922_924delAGCinsT	p.Ser308*	Nonsense	7
11	1324delA	c.1205delA	p.Glu402Glyfs	Frameshift	1
11	1630dupG	c.1511dupG	p.Lys505*	Nonsense	1
11	2508_2509delGA	c.2389_2390delGA	p.Glu797Thrfs	Frameshift	1
11	2682C>T	c.2563C>T	p.Gln855*	Nonsense	1
11	3415delC	c.3296delC	p.Pro1099Leufs	Frameshift	1
11	3561delG	c.3442delG	p.Glu1148Argfs	Frameshift	3
11	3746dupA	c.3627dupA	p.Glu1210Argfs	Frameshift	5
11	3877_3878delinsA	c.3758_3759delinsA	p.Ser1253*	Nonsense	1
11	3932dupT	c.3813dupT	p.Asn1272*	Nonsense	1
12	4246_4247delCA	c.4127_4128delCA	p.Thr1376fs	Frameshift	1
13	4372delT	c.4253delT	p.Leu1418*	Nonsense	1
17	5136delCAC	c.5017_5019delCAC	p.His1673del	Inframe variant	1
18	5199G>T	c.5080G>T	p.Glu1694*	Nonsense	2
20	5385C>T	c.5266C>T	p.Gln1756*	Nonsense	1
22	5458T>C	c.5339T>C	p.Leu1780Pro	Missense	1
23	5564G>A	c.5445G>A	p.Trp1815*	Nonsense	1
23	5586+1G>A	c.5467+1G>A	Skipping of exon 23	Splice donor variant	1

BIC, Breast Cancer Information Core; HGVS, Human Genome Variation Society.
