# Supplementary material for: Effect of BRCA mutational status on survival outcome in advanced-stage high-grade serous ovarian cancer
Source: J Ovarian Res. 2019 May 7;12:40. doi: 10.1186/s13048-019-0511-7 (PMC6505247; doi:10.1186/s13048-019-0511-7)
Supplement: Supplementary file 1 — Figure S1. Comparisons of survival outcomes for all patients according to the primary treatment strategy. (A) Overall survival. (B) Progression-free survival. Figure S2. Comparisons of survival outcomes among the patients with germline BRCA1 mutations according to the three domains: 1) the N-terminal Really Interesting New Gene (RING) domain (exons 2–7); 2) Exons 11–13, that covers over 65% of the sequence of BRCA1; and 3) the BRCA1 C-terminal (BRCT) domain (exons 16–24). (A) Overall survival. (B) Progression-free survival. Table S1. Personal and familial histories of patients at diagnosis of epithelial ovarian cancer. Table S2. Clinicopathological characteristics of patients according to the primary treatment strategy. Table S3. Factors associated with survival outcomes in primary debulking surgery group. Table S4. Factors associated with survival outcomes in neoadjuvant chemotherapy group. Table S5. Deleterious BRCA1 gene mutations in this study. (ZIP 580 kb) [file 13048_2019_511_MOESM1_ESM.zip › Supplementary Table 2.docx]

| **Supplementary Table 2.** Clinicopathological characteristics of patients according to the primary treatment strategy | | | | | | |
| --- | --- | --- | --- | --- | --- | --- |
| Characteristics | *BRCA* mutation | | | *BRCA* wild-type | | |
|  | PDS  (*n*=32, %) | NAC  (*n*=19, %) | *P* | PDS  (*n*=47, %) | NAC  (*n*=30, %) | *P* |
| Age, years |  |  |  |  |  |  |
| Mean ± SD | 54.6 ± 10.5 | 54.8 ± 9.2 | 0.966 | 56.1 ± 9.5 | 59.7 ± 11.6 | 0.143 |
| BMI, kg/m2 |  |  |  |  |  |  |
| Mean ± SD | 23.2 ± 3.8 | 23.1 ± 3.7 | 0.948 | 23.6 ± 3.2 | 23.5 ± 3.8 | 0.901 |
| CA-125, IU/ml |  |  |  |  |  |  |
| Median (range) | 695.5 (34.9-9926.0) | 1390.0 (194.0-7846.0) | 0.119 | 764.0 (38.1-4452.0) | 1946.0 (13.0-10000.0) | 0.011 |
| FIGO stage |  |  | 0.009 |  |  | 0.050 |
| III | 28 (87.5) | 10 (52.6) |  | 28 (59.6) | 11 (36.7) |  |
| IV | 4 (12.5) | 9 (47.4) |  | 19 (40.4) | 19 (63.3) |  |
| Residual tumor after PDS/IDS |  |  | 0.639 |  |  | 0.378 |
| Optimal debulking | 28 (87.5) | 18 (94.7) |  | 37 (78.7) | 26 (86.7) |  |
| Suboptimal debulking | 4 (12.5) | 1 (5.3) |  | 10 (21.3) | 4 (13.3) |  |
| Abbreviations: BMI, body mass index; CA-125, cancer antigen 125; FIGO, International Federation of Gynecology and Obstetrics; PDS, primary debulking surgery; NAC, neoadjuvant chemotherapy; IDS, interval debulking surgery; SD, standard deviation. | | | | | | |
